# The Expression of Cytokine Profiles and Related Receptors in Idiopathic Inflammatory Myopathies

**DOI:** 10.3389/fphar.2022.852055

**Published:** 2022-04-20

**Authors:** Junyu Zhou, Lijuan Zhao, Yizhi Xiao, Shasha Xie, Ying Long, Yu Wei, Qiming Meng, Xiaojing Li, Hui Luo, Honglin Zhu

**Affiliations:** ^1^ The Department of Rheumatology and Immunology, Xiangya Hospital of Central South University, Changsha, China; ^2^ Provincial Clinical Research Center for Rheumatic and Immunologic Diseases, Xiangya Hospital, Changsha, China; ^3^ National Clinical Research Center for Geriatric Disorders, Xiangya Hospital, Changsha, China,

**Keywords:** idiopathic inflammatory myopathies, cytokine profiles, receptors, IL18R1, cytokines

## Abstract

**Background:** Cytokines play a vital role in the pathogenesis of idiopathic inflammatory myopathies (IIMs). Here, we investigated the expression of serum cytokine profiles in untreated IIMs and their correlations with clinical indicators, and further studied the expression of related cytokines receptors in IIMs.

**Methods:** The Human 48-Plex Luminex assay for cytokines was performed in the serum of IIMs, including 93 untreated and 18 follow-up (39 samples) patients, and 32 healthy controls (HC). Mann-Whitney U test with bonferroni adjusted was used to identify the differentially expressed cytokines among groups. Celltalker software was used to identify the receptors of differentially expressed cytokines. The expression of receptors was further validated by published GEO datasets (muscle, blood and skin), RT-qPCR, western blot and flow cytometry.

**Results:** The serum levels of Eotaxin, IL7, IL18, IP10, MCP1, MCSF, MIG and SCGFβ were elevated in the 93 untreated patients. Except for IL7, all other cytokines were decreased after treatment and their levels were positively correlated with clinical indices such as LDH, ESR, CRP, ALT, IgA, AST and IgG while negatively correlated with albumin and MMT8. According to the serum myositis-specific antibodies (MSAs), patients were classified into three groups: anti-ARS (Jo-1, OJ, EJ, PL7, PL12), anti-MDA5 positive, and anti-TIF1γ positive. Compared with HC, the levels of IP10 and MIG were increased in three groups. Moreover, IL18 and MSCF were increased in anti-ARS patients, and CTACK, Eotaxin, IL1Rα, IL7, IL18, MCP1, MCP3, MCSF and SCGFβ were elevated in anti-MDA5 patients. Twenty receptors of the 8 differentially expressed cytokines were matched by celltalker software, among them, IL18R1 and CCR1 were up-regulated in blood, muscle and skin of IIMs from the analysis of GEO published datasets. RT-qPCR and western blot further validated IL18R1 was upregulated in the muscle tissues of dermatomyositis. The number of IL18R1^+^CD4^+^ cells was increased while IL18R1^+^CD8^+^ cells was decreased in peripheral blood of anti-MDA5 patients.

**Conclusion:** This study showed that cytokine profiles were significantly changed in IIMs, and different MSA groups had unique cytokine expression patterns. The levels of some cytokine were correlated with clinical indices. The IL18 receptor IL18R1 might play important roles in IIMs.

## Introduction

Idiopathic inflammatory myopathies (IIMs) are a group of rare autoimmune diseases that affect the muscle, and organs such as the skin and lungs (Lundberg et al., 2018). The common subtypes of IIMs are dermatomyositis (DM), polymyositis (PM), inclusion body myositis (IBM), and amyopathic dermatomyositis (ADM) (Lundberg et al., 2017). However, some clinical or histopathological manifestations among these subgroups overlap. Recently, myositis-specific antibodies (MSAs), which are more associated with myositis clinical manifestations are used for diagnosis and classification of IIMs.

MSAs include the anti-melanoma differentiation gene 5 (MDA5), anti-transcription intermediary factor 1-γ (TIF1γ), anti-aminoacyl transfer RNA synthetases (ARS) (Jo-1, EJ, OJ, PL7, PL12, KS) and anti-signal recognition particle (SRP) antibodies (Lundberg et al., 2018; Mammen et al., 2020). These four classes of MSAs present different clinical manifestations. Anti-ARS positive and anti-MDA5 positive IIMs are often associated with interstitial lung disease (ILD) (Satoh et al., 2017; Allenbach et al., 2020), anti-TIF1-γ positive IIMs are associated with malignancy (Hida et al., 2016), and anti-SRP positive IIMs are associated with severe muscle weakness (Dalakas, 2016).

The pathogenesis of IIMs remains elusive. In DM, infiltration of B cells, CD4^+^ T cells, and plasmacytoid dendritic cells are found in the perimysial and endomysial areas, while in PM, CD8^+^ T cells surround nonnecrotic muscle fibers (Ernste and Reed, 2013; Dalakas, 2015). These phenomena indicate that immune cells play an important role in the pathogenesis of IIMs. Cytokines such as IFNs and TNF-α, primarily secreted by these immune cells, are keys to a cascade of inflammatory mediators, and are observed in patients with chronic inflammation and necrosis of muscle fiber (Greenberg et al., 2005; Schmidt et al., 2008). In addition, increased Eotaxin, IL10, IP10, and MCP1 are found in anti-MDA5 patients with interstitial lung disease (Asakawa et al., 2020); In patients with rapidly progressive interstitial lung disease (RP-ILD), IFNγ, IL1β and IL12 levels are considerably elevated (Ishikawa et al., 2018). Serum IFN-β and CXCL10 levels are correlated with the Cutaneous Dermatomyositis disease Area and Severity Index score (Chen et al., 2018). These studies indicate that cytokines play vital roles in the immune mechanism of IIMs.

However, there are no reports about the changes of cytokine profiles in different untreated IIMs MSAs groups, and researches studying the changes of receptors for differentially expressed cytokines are lacking. Herein, we aimed to determine the levels of cytokine profiles in untreated IIMs patients and in the MSAs subtypes of IIMs. Meanwhile, we analyzed the correlations between cytokines and clinical data and further investigated the expression of cytokine receptors in IIMs.

## Materials and Methods

### Patients and Healthy Controls

Patients were retrospectively recruited, and admitted to the Department of Rheumatology and Immunology in Xiangya Hospital Central South University (Changsha, Hunan, China) from 2018 to 2020. Patients were older than 18 years at age of onset, and met Bohan and Peter’s criteria (Bohan and Peter, 1975). Patients with co-occurrence of other autoimmune diseases (e.g., rheumatoid arthritis, Systemic lupus erythematosus, etc.) and infection were excluded. Thereinto, Sera for the multi-cytokines assay were obtained from 93 untreated IIMs patients, 18 follow-up IIMs patients, and 32 healthy controls (HC). For real-time quantitative polymerase chain reaction (RT-qPCR) analysis, the muscle cDNA was obtained from 19 DM patients and 16 HC. For western blot analysis, muscle protein included 8 DM and 5 HC samples. For flow cytometry, 19 untreated IIMs cases (9 anti-MDA5 positive), 24 treated IIMs cases and 18 HC were included. This study was approved by the ethics committee at Xiangya Hospital Central South University. All participants provided written informed consent.

### Evaluation of Clinical Data

Skin evaluation for heliotrope rash, Gottron’s sign or papule, and muscle evaluation for myosalgia, myasthenia, and manual muscle testing 8 (MMT8) were conducted by the same physician. Laboratory examination creatine kinase (CK), glutamic-pyruvic transaminase (ALT), glutamic-oxalacetic transaminase (AST), high density lipoprotein (HDL), low density lipoprotein (LDL), lactate dehydrogenase (LDH), erythrocyte sedimentation rate (ESR), and C-reactive protein (CRP) were obtained from medical records. ILD was assessed by high-resolution chest CT (HRCT).

### Assessment of MSAs

The MSAs in serum samples (anti-ARS (Jo-1, EJ, OJ, PL-7, PL-12), anti-MDA5, and anti-TIF1γ) were detected using commercial kits (EUROIMMUN, Germany).

### Measurement of Cytokines

All serum samples from IIMs patients and HCs were stored at −80°C without repetitive freezing and thawing. The levels of cytokines (CTACK, Eotaxin, Basic FGF, GCSF, GMCSF, GROα, HGF, IFNα2, IFNγ, IL1α, IL1β, IL1ra, IL2, IL-2Rα, IL3, IL4, IL-5, IL6, IL7, IL8, IL9, IL10, IL12(P40), IL12 (P70), IL13, IL15, IL16, IL17, IL18, IP10, LIF, MCP1, MCP3, MCSF, MIF, MIG, MIP1α, MIP1β, β-NGF, PDGFBB, RANTES, SCF1α, SCFGβ, SDF1α, TNFα, TNFβ, TRAIL, VEGF) were determined by Wayen Biotechnology (Shanghai, China) using the Human 48-Plex Luminex assay.

### Gene Expression Omnibus (GEO) Datasets Analysis of Receptors Corresponding to Cytokines

The R package DESeq2 was used to identify significant DE genes (DEG) in GSE125977 (whole blood, RNAseq, Illumina HiSeq (4,000) and (7 DM patients and 5HC) datasets (*p* < 0.05), while the R package limma was used to identify significant DEG in the GSE143323 (muscle, RNAseq, Illumina HiSeq 3000, 39 DM patients and 20 HC), GSE142807 (skin, microarray, Affymetrix Human Gene 2.1 ST Array, 43 DM patients and 5 HC) and GSE128314 (skin, microarray, Affymetrix Human Transcriptome Array 2.0, 3 anti-MDA5 positive DM patients and 5 HC) datasets (*p* < 0.05).

### Real-Time Quantitative Polymerase Chain Reaction Analysis

Muscle tissues were obtained from the left biceps of IIMs patients and from HC who underwent joint replacement. Total RNA was extracted from muscle tissue using TRIzol reagent according to the manufacturer’s instructions. To detect the expression of IL18R1, RT-qPCR was performed. RNA concentration and quality were determined using a QS3000 spectrophotometer. RT-qPCR was performed using gene-specific primers with SYBR Green (SYBR Premix Ex Taq RT-PCR kit; Takara) on a 7,500 Real-Time PCR System (Applied Biosystems, Waltham, MA, United States ). The sequences of primers used in the present study were as follows: IL18R1, forward: 5′-GGA​GGC​ACA​GAC​ACC​AAA​AGC​T-3′ and; IL18R1, reverse: 5′-AGG​CAC​ACT​ACT​GCC​ACC​AAG​A-3′. β-actin was used as an internal control and the relative expression levels were determined by the 2 ˗∆∆ CT method.

### Western Blot Analysis

Total protein of muscle tissues was extracted using RIPA lysate (Beyotime, Shanghai, China). BCA protein detection kits (Thermo Fisher Scientific, Waltham, MA, United States ) were used to determine the protein concentrations of each group. The same amounts of protein were separated using 8% SDS-PAGE and transferred to a polyvinylidene fluoride (PVDF) membrane. Thereafter, PVDF membranes were sealed with 5% skim milk powder in 0.1% Tween’s Tris-buffer saline for 2 h, and subsequently incubated with specific primary antibodies against IL18R1 (DF7059, Affinity, China), vinculin (ab219649, Abcam, China) overnight at 4°C. Thereafter, the anti-rabbit IgG enzyme-conjugated secondary antibody (Proteintech, Wuhan, China) was incubated at room temperature for 1 h. Enhanced chemiluminescence (Bio-RAD, Hercules, CA, United States ) was used to detect western blot results.

### Flow Cytometry

Fresh whole blood samples were obtained from IIMs patients and HC and stored in EDTA tubes, of which 100 µl was placed into flow tubes. BB700-conjugated anti-human CD4, APC-Cy7-conjugated anti-human CD8, and PE-conjugated anti-human IL18R1 (all from BD Pharmingen, United States ) were immunolabelled with the samples at 4°C for 20 min. Thereafter, the mixture underwent erythrocyte lysis, centrifuge washing, and flow cytometry (BD Biosciences). The results were analyzed using FlowJo v10.

### Statistical Analysis

R (version 4.0.2) was used to perform statistical analysis. For clinical data, non-normally distributed data are presented as median (interquartile range); otherwise, normally distributed data are presented as mean ± standard deviations. Continuous variables were assessed using the Kruskal–Wallis H test followed by the Mann-Whitney U test for each of the two groups (*p*-values were adjusted using the “Bonferroni” method) and categorical variables were analyzed using Fisher’s exact test (*p*-values for multiple comparison were also adjusted using the “Bonferroni” method). The Mann–Whitney *U* test was used for each of the two groups (*p*-values were adjusted using the “Bonferroni” method during the multiple comparisons test) when comparing the cytokine levels. Correlation coefficients were established by employing Spearman’s correlation coefficients. For RT-qPCR, western blot, and flow cytometry analysis, an unpaired *t*-test was used for comparison between the two groups. The following symbols were used: *p < 0.05, **p < 0.01.

## Results

### Clinical Characteristics of all Patients

Ninety-three untreated IIMs (71 females) were enrolled in this study (Table 1). The average onset age was 52.28 ± 12.90 years and the median disease duration was 4 months. For MSA, the number of anti-ARS (Jo-1, OJ, EJ, PL7, PL12), anti-MDA5, anti-TIF1γ, anti-SRP, and Overlap autoantibodies were 24, 27, 15, 5, and 22 respectively. Of the participants, 65.9% were diagnosed with ILD. The frequency of clinical features such as heliotrope rash, Gottron’s sign or papule, myosalgia, and myasthenia was 24.7, 55.9, 46.2, and 74.2%, respectively. The median of MMT8 was 72.00. A total of 39 samples were collected from 18 follow-up patients (Supplementary Table S1). In terms of treatments, the top three were glucocorticoid (98.9%), tacrolimus (53.8%), and cyclophosphamide (22.6%) (Supplementary Table S1). Baseline clinical data among anti-ARS positive, anti-MDA5 positive and anti-TIF1γ positive IIMs were compared (Table 2). There were no differences in sex, age, muscle characteristics, ALT, AST, HDL, LDH, ESR, or CRP among the three groups. Anti-ARS positive and anti-MDA5 positive groups were more likely to incorporate ILD. The proportion of skin characteristics including heliotrope sign and Gottron’s sign or papule, were higher in anti-MDA5 positive and anti-TIF1γ positive groups. For LDL and CK levels, anti-ARS positive and anti-TIF1γ positive groups were higher than anti-MDA5 positive group. Differences among the three groups were noted for ferritin.

**TABLE 1:** Clinical characteristics of untreated IIMs patients.

	*n* = 93
Sex, female (%)	71 (76.3)
Age, year	52.28 ± 12.90
Disease duration, months	4.00 [2.00, 6.00]
Specific-antibody	
ARS (%)	24 (25.8)
MDA5 (%)	27 (29.0)
TIF1γ (%)	15 (16.1)
SRP (%)	5 (5.4)
Overlap (%)	22 (23.7)
ILD (%)	58 (65.9)
Skin characteristics	
Heliotrope rash (%)	23 (24.7)
Gottron’s sign or papule (%)	52 (55.9)
Muscle characteristics	
Myosalgia (%)	43 (46.2)
Myasthenia (%)	69 (74.2)
MMT8	72.00 [64.00, 80.00]

Non-normally distributed data are given as median (interquartile range) otherwise normally distributed data are given as mean ± standard deviation.

**TABLE 2:** Comparison of clinical information among anti-ARS positive, anti-MDA5 positive and anti-TIF1γ positive untreated IIMs patients.

	ARS (*n* = 24)	MDA5 (*n* = 27)	TIF1γ (*n* = 15)	*p*
Sex, female (%)	20 (83.3)	20 (74.1)	11 (73.3)	0.749
Age, year	52.29 ± 12.66	46.67 ± 9.99	53.53 ± 17.62	0.173
ILD, *n* (%)	18 (81.8)	18 (72.0)	1 (6.7)	<0.001^a^ ^,^ ^b^
Skin characteristics				
Heliotrope sign, *n* (%)	0 (0.0)	12 (44.4)	6 (40.0)	<0.001^a^ ^,^ ^c^
Gottron’s sign or papule, *n* (%)	8 (33.3)	21 (77.8)	12 (80.0)	0.001^a^ ^,^ ^c^
Muscle characteristics				
Myosalgia, *n* (%)	9 (37.5)	14 (51.9)	7 (46.7)	0.661
Myasthenia, *n* (%)	16 (66.7)	18 (66.7)	12 (80.0)	0.7
MMT8	78.00 (70.00, 80.00)	72.00 (64.00, 77.00)	73.00 (62.50, 79.00)	0.157
ALT (U/L)	50.15 (29.00, 93.42)	40.90 (23.00, 76.35)	26.00 (19.25, 56.00)	0.331
AST (U/L)	54.10 (32.25, 78.45)	55.30 (43.60, 104.15)	53.70 (32.60, 86.60)	0.787
HDL (mmol/L)	0.81 (0.68, 1.04)	0.74 (0.57, 0.89)	0.96 (0.79, 1.19)	0.063
LDL (mmol/L)	2.87 (2.62, 3.39)	2.24 (1.84, 2.63)	3.07 (2.54, 3.41)	0.002^b^ ^,^ ^c^
LDH (U/L)	413.00 (321.50, 514.75)	414.75 (314.25, 476.50)	333.00 (311.00, 362.50)	0.173
CK (U/L)	976.10 (204.62, 1690.17)	111.55 (57.30, 209.70)	279.00 (161.05, 475.55)	<0.001^b^ ^,^ ^c^
ESR (mm/h)	54.00 (30.50, 83.25)	61.00 (30.75, 83.25)	46.00 (35.00, 56.50)	0.581
CRP (mg/L)	7.12 (2.30, 21.65)	4.93 (3.31, 8.29)	3.08 (1.89, 5.27)	0.159
Ferritin (μg/L)	311.50 (140.98, 723.15)	623.20 (242.95, 1612.00)	288.60 (179.15, 512.85)	0.038

Non-normally distributed data are given as median (interquartile range); otherwise normally distributed data are given as mean ± standard deviation. Continuous variables were analyzed using the Kruskal-Wallis H test followed by the Mann–Whitney U test for each of two groups (*p*-values were adjusted using the “Bonferroni” method during the multiple comparisons test) and categorical variables were analyzed by Fisher’s exact test (*p*-value for multiple comparison were also adjusted using “Bonferroni” method).

a ARS vs TIF1γ, *p* < 0.05.

b MDA5 vs TIF1γ, *p* < 0.05.

c ARS vs MDA5, *p* < 0.05.

### Cytokine Profiles and Correlation Analysis in IIMs Patients

**FIGURE 1:**
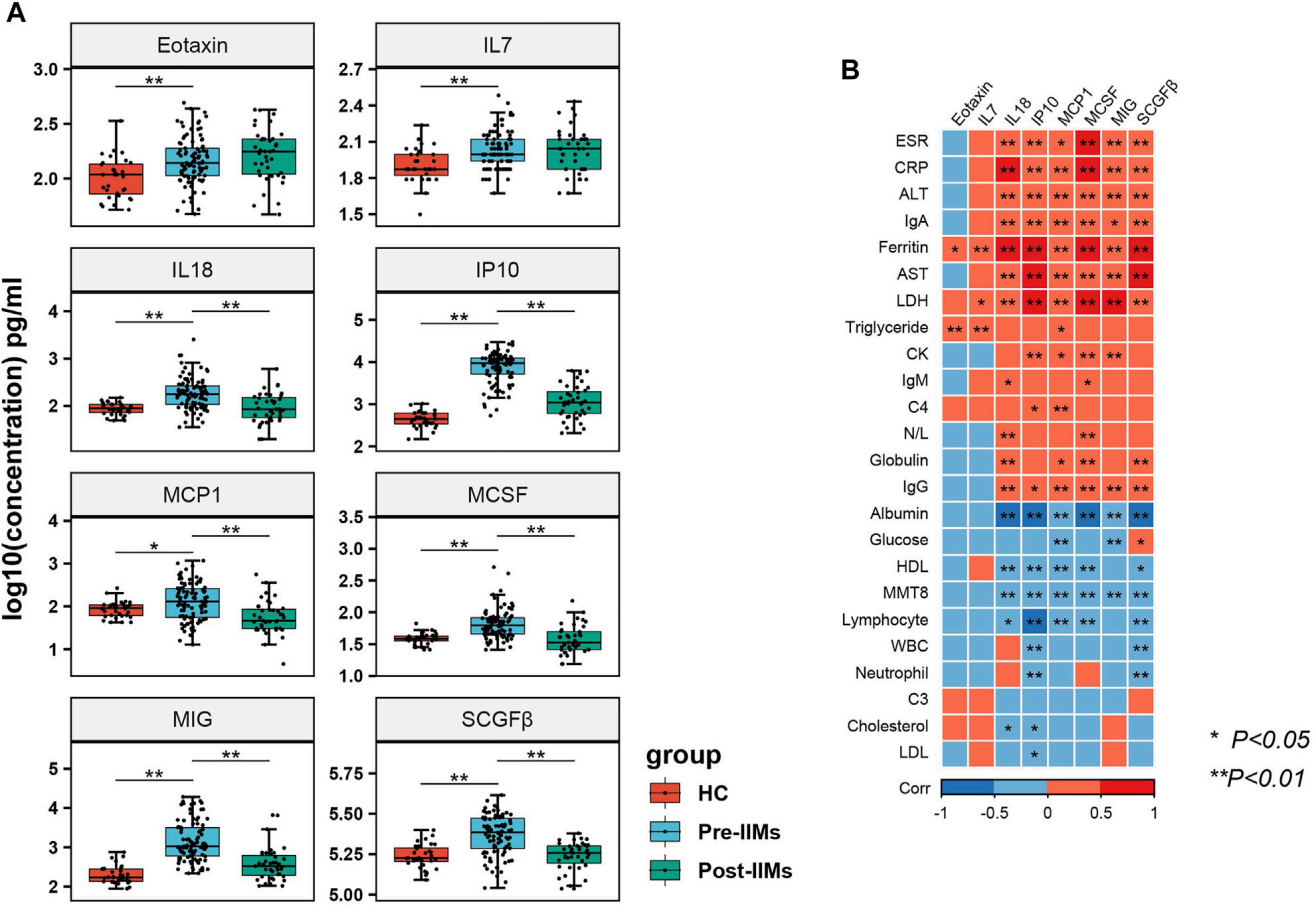
The expression of cytokine profiles and correlation analysis in IIMs. **(A)** Comparison of cytokines expressions among IIMs patients (before and after treatment) and healthy controls (HC). The Human 48-Plex Luminex assay for cytokines was performed in the serum of IIMs patients, including 93 untreated and 18 follow-up (39 samples) patients, and 32 healthy controls. Mann-Whitney U test was used to compare the marked groups. *p < 0.05, **p < 0.01. Pre-IIMs: IIMs before treatment; Post-IIMs: IIMs after treatment (glucocorticoids and other immunosuppressive therapies). **(B)** Correlation analysis among cytokines and clinical indices in all enrolled IIMs patients. Statistical analyses were performed using Spearman’s correlation coefficients. *p < 0.05, **p < 0.01. N/L: Ratio of neutrophils to lymphocytes.

Cytokines between untreated IIMs, treated IIMs and HCs were analyzed (Figure 1A). The levels of Eotaxin, IL7, IL18, IP10, MCP1, MCSF, MIG and SCGFβ were significantly increased in untreated IIMs than in HC. After treatment, the levels of IL18, IP10, MCP1, MCSF, MIG, and SCGFβ were significantly decreased. Correlation analysis (Figure 1B) showed that all of 8 cytokines had a distinct correlation with ferritin, especially IL18, IP10, MCP1, and MCSF. In addition, IL7 was positively correlated with LDH; IL18, IP10, MCP1, MCSF, MIG and SCGFβ were positively correlated with LDH, ESR, CRP, ALT, IgA, AST and IgG and negatively correlated with albumin and MMT8.

### Comparison of Cytokines Among anti-ARS Positive, anti-MDA5 Positive, anti-TIF1γ Positive IIMs and HC

**FIGURE 2:**
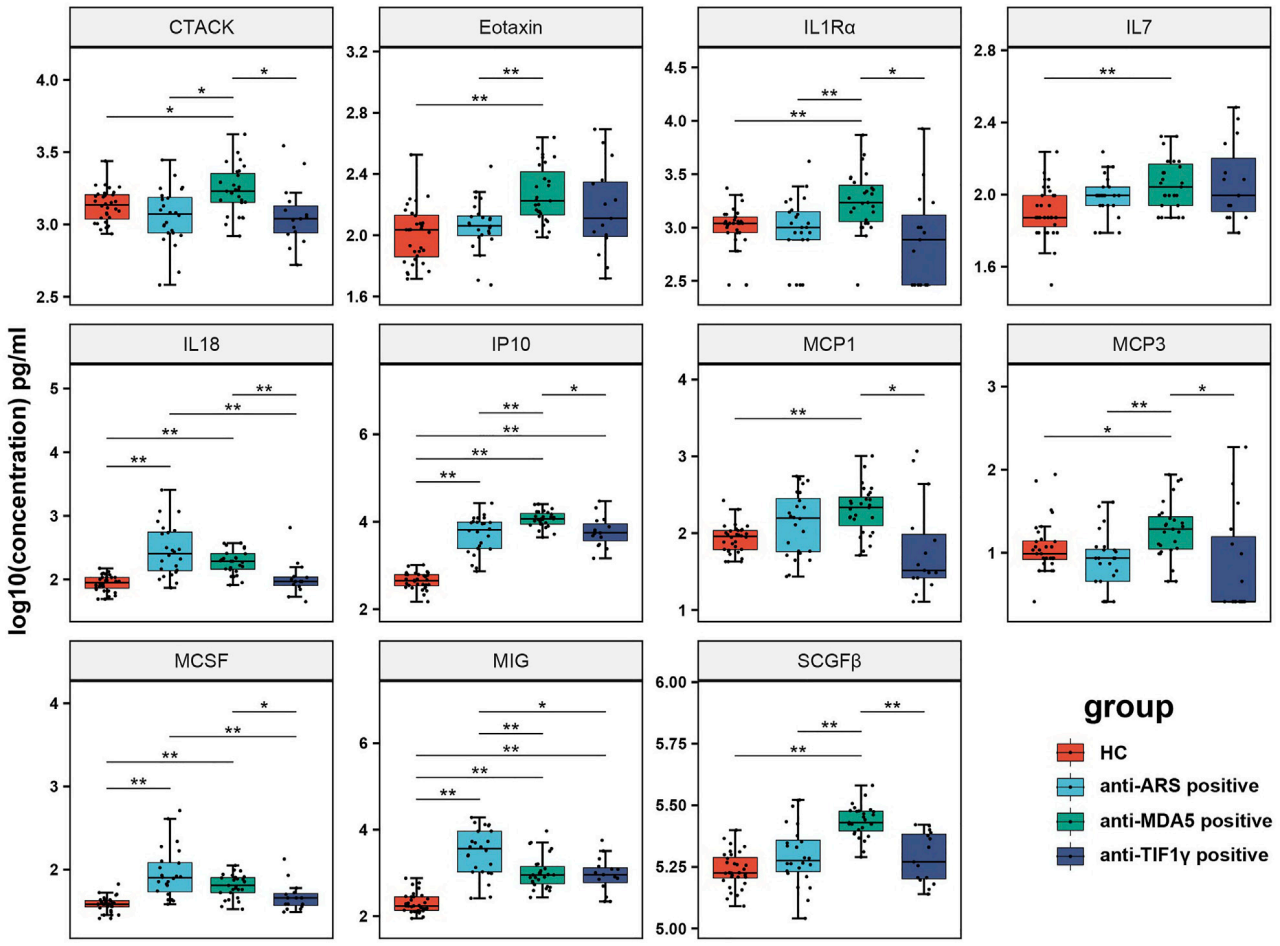
Comparison of cytokines among anti-ARS positive, anti-MDA5 positive, anti-TIF1γ positive IIMs patients and HC. The Human 48-Plex Luminex assay for cytokines was performed in the serum of different MSAs IIMs groups (24 anti-ARS positive, 27 anti-MDA5 positive, 15 anti-TIF1γ positive). Mann-Whitney U test was used to compare the marked groups (*p*-values were adjusted using the “Bonferroni” method during the multiple comparisons test). *p < 0.05 and **p < 0.01.

Cytokines between IIMs groups and HC were analyzed (Figure 2). A significantly increased expression of IL18, IP10, MSCF, and MIG was observed in the anti-ARS positive group compared with HC. When comparing the anti-MDA5 positive group and HC, elevated cytokines in the anti-MDA5 positive group were CTACK, Eotaxin, IL1Rα, IL7, IL18, IP10, MCP1, MCP3, MCSF, MIG and SCGFβ. However, only IP10 and MIG were increased in the anti-TIF1γ positive group when compared with HC. Upon investigating the expression levels of these 11 cytokines in the three disease groups, we found that the levels of CTACK, Eotaxin, IL1Rα, IP10, MCP3, and SCGFβ were higher in the anti-MDA5 positive group than those in the anti-ARS positive group; however, the level of MIG was decreased. Upon comparison of the anti-ARS positive group and the anti-TIF1γ positive group, we found that only IL18 and MCSF were increased in the anti-ARS positive group. However, CTACK, IL1Rα, IL18, IP10, MCP1, MCP3, MCSF and SCGFβ were elevated in the anti-MDA5 positive as compared to the anti-TIF1γ positive group.

### Expression of Receptors of Differentially Expressed Cytokines

**FIGURE 3:**
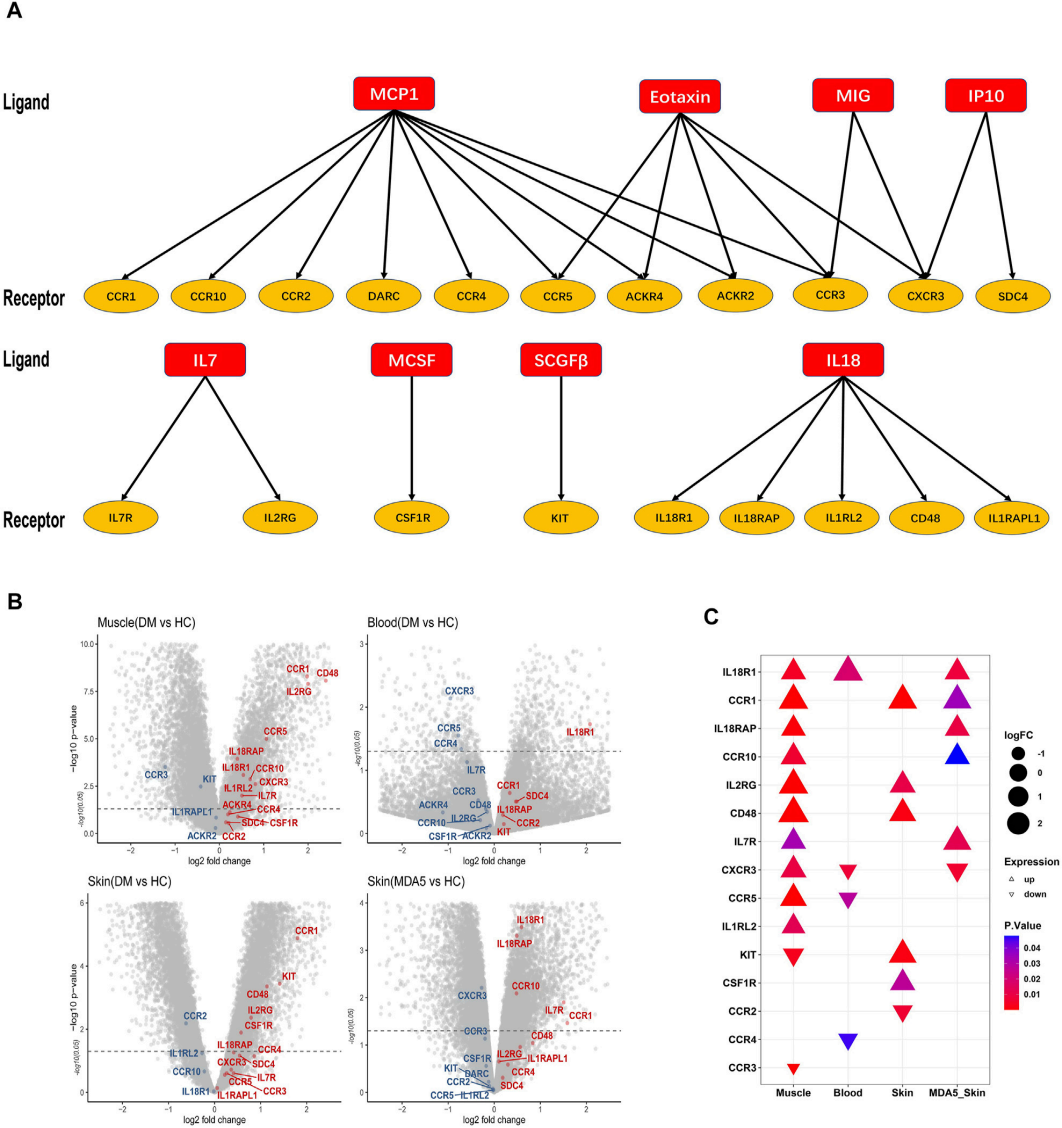
The expression of receptors of the differentially expressed cytokines. **(A)** The ligand-receptor database (ramilowski_pairs) was analyzed with the R package celltalker to identify a total of 20 receptors corresponding to the 8 differentially expressed cytokines. **(B)** The volcano plots showed the expression of 20 selected receptors in four different databases (GSE143323, GSE125977, GSE142807, and GSE128314). Red color: up-regulated expression; Blue color: down-regulated expression. **(C)** Fifteen significant (*p* < 0.05) receptors were identified from the four selected GEO databases.

To further explore the expression of the receptors for the 8 differentially expressed cytokines (Eotaxin, IL7, IL18, IP10, MCP1, MCSF, MIG and SCGFβ) in inflammatory myopathy, we used the ligand-receptor database (ramilowski_pairs) in the R package celltalker. Twenty receptors of the 8 cytokines were identified (Figure 3A). Then we validated the expression of these 20 receptors in public GEO datasets, including muscle (GSE143323), blood (GSE125977), skin (GSE142807), and anti-MDA5-positive_skin (GSE128314) in DM patients (Figure 3B). Fifteen significant (*p* < 0.05) receptors (IL18R1, IL18RAP, CCR1, CCR10, IL2RG, CD48, IL7R, CXCR3, CCR5, IL1RL2, KIT, CSF1R, CCR2, CCR4 and CCR3) were observed in one or more datasets. Significantly increased levels of IL18R1 and CCR1 were observed in the three datasets (Figure 3C).

### Expression of IL18R1 in Muscle Tissues and Peripheral Blood Cells of IIMs Patients

**FIGURE 4:**
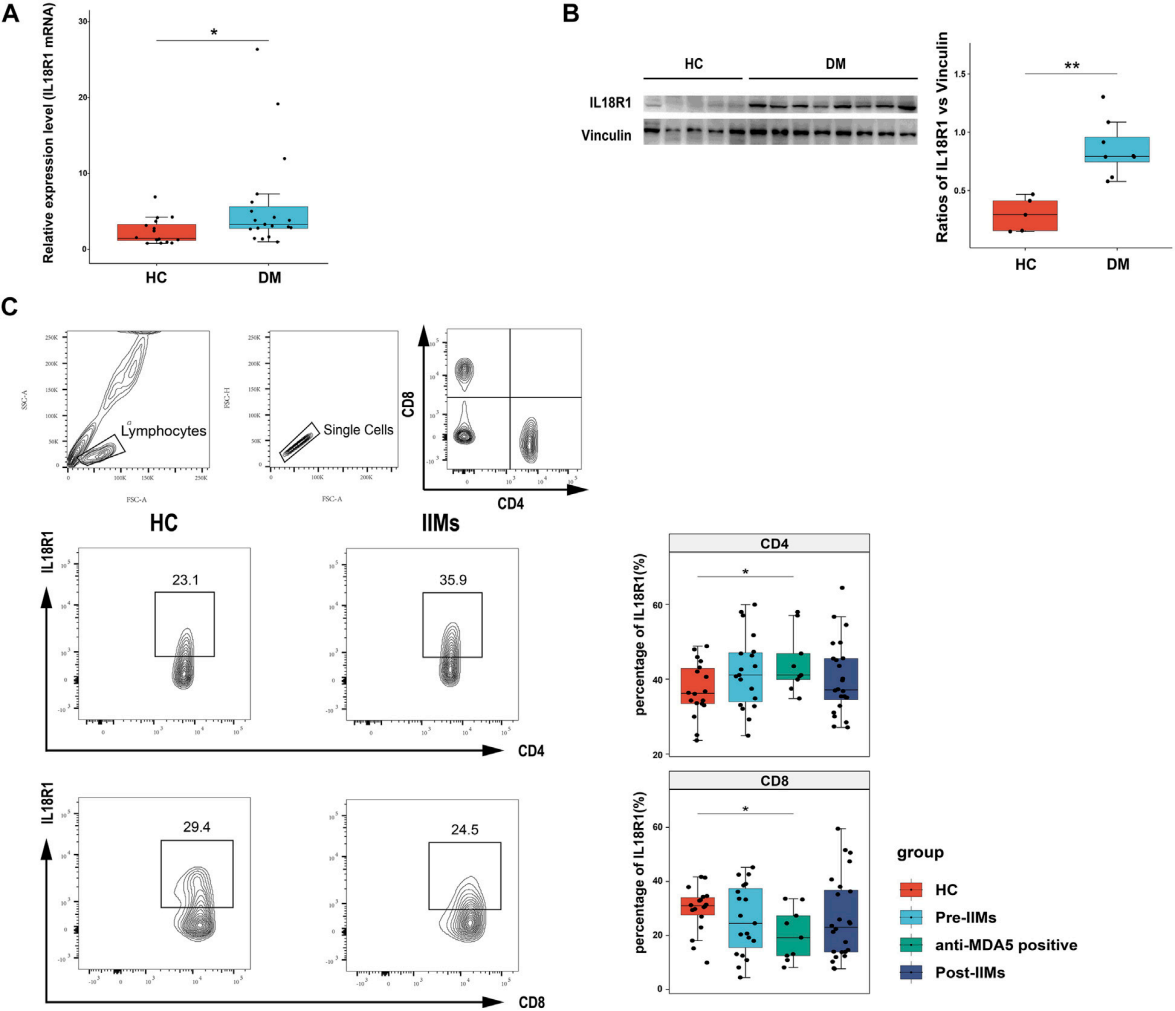
Expression of IL18R1 in the muscle tissues and blood of IIMs patients. **(A)** The expression of IL18R1 mRNA in the muscle tissues of 19 DM patients and 16 HC. **(B)** Western blot showed the expression of IL18R1 protein in muscle tissues of 8 DM patients and 5 HC. **(C)** Flow cytometry analysis the expression of IL18R1 on lymphocytes among 19 untreated IIMs (9 anti-MDA5 positive) patients, 24 treated IIMs patients and 18 HC. Unpaired *t*-test was used for comparison. Pre-IIMs: IIMs before treatment; Post-IIMs: IIMs treatment. *p < 0.05, **p < 0.01.

The level of IL18 was elevated in patients with inflammatory myopathy. Through previous analysis, IL18R1, one of the receptors associated with IL18, was upregulated in the public database (muscle, blood, and anti-MDA5-related skin) we selected; thus, we further studied the expression of IL18R1 in muscle tissues and peripheral blood cells of IIMs patients. RT-qPCR results showed that the expression of IL18R1 was significantly elevated in muscle tissue in the DM group (Figure 4A). Western blot analysis also confirmed that the level of IL18R1 was increased in DM muscle tissues (Figure 4B). Alternatively, flow cytometry was performed on peripheral blood from both myositis patients and HC (Figure 4C). No significant differences in IL18R1 in CD4 or CD8 cells were observed in all untreated IIMs patients compared with HC. The percentage of IL18R1^+^CD4^+^ cells (the proportion of IL18R1 expression in total CD4^+^ cells) in MDA5^+^DM patients was higher than that in HC, while IL18R1^+^CD8^+^ cells in MDA5^+^DM patients was lower than that in HC. Comparison between the before treatment group and the after treatment group showed no difference.

## Discussion

In our study, 8 serum cytokines were elevated in IIMs, including Eotaxin, IL7, IL18, IP10, MCP1, MCSF, MIG and SCGFβ. Moreover, according to the MSAs classification, we found that the expression of CTACK, Eotaxin, IL1Rα, IL7, IL18, IP10, MCP1, MCP3, MCSF, MIG and SCGFβ were differentially expressed in anti-ARS positive, anti-MDA5 positive, anti-TIF1γ positive IIMs compared with HC groups. These results were partially consistent with those of previous reports (Gono et al., 2010; Gono et al., 2014; Zou et al., 2016; Chen et al., 2018; Bai et al., 2020; Sugimoto et al., 2021). In addition, serum SCGFβ and CTACK were first found to be increased in IIMs and anti-MDA5 positive patients, respectively. SCGFβ, one of two isoforms of hematopoietic growth factor, plays a role in promoting hematopoiesis and as an indicator of hematopoietic recovery; it has been increasingly reported in tissues and organs other than the bone marrow (Hiraoka et al., 1997; Mio et al., 1998; Ito et al., 2003; Wang et al., 2013; Schiro et al., 2015; Chen et al., 2020). The increase of SCGFβ in IIMs might be related to granulocyte/macrophage colony-promoting activity, in that macrophages have been found infiltrating muscle species, especially in DM (Preusse et al., 2012; Day et al., 2017). CTACK, a skin-associated chemokine, has been demonstrated to attract memory T cells (Morales et al., 1999). This phenomenon has been reported in previous studies on skin-related diseases, such as psoriasis (Campanati et al., 2007), oral lichen planus (Marshall et al., 2018), atopic dermatitis (Machura et al., 2012), and systemic sclerosis (Hayakawa et al., 2005). In our study, the serum CTACK was primarily overexpressed in anti-MDA5 positive DM patients, which might be associated with distinctive skin lesions of patients. We also found the levels of the 8 differentially expressed cytokines (Eotaxin, IL7, IL18, IP10, MCP1, MCSF, MIG and SCGFβ) were decreased after glucocorticoids or immunosuppression treatment, and some clinical indicators such as ferritin, ESR, CRP, CK, LDH, ALT, and AST were decreased after treatment (data no shown). These results indicated that the 8 cytokines can reflect the inflammatory immune status, which can be used as potential biomarkers. However, due to the limited number of followed-up patients and the short follow-up time, more relevant assessments of therapeutic efficacy remain to be discussed in future.

In the correlation analysis, 6 or more of the 8 upregulated cytokines (Eotaxin, IL7, IL18, IP10, MCP1, MCSF, MIG and SCGFβ) positively correlated with ferritin, LDH, ESR, CRP, ALT, IgA, AST, and IgG, and negatively correlated with albumin and MMT8. Inflammatory and immune-related indicators especially ESR and CRP are reported to reflect the degree of disease activity (Go et al., 2016; Gono et al., 2021). Previous reports have shown hyperferritinemia in RP-ILD in anti-MDA5 positive patients (Gono et al., 2010; Nakashima et al., 2010), and LDH is treated as a poor prognostic factor for DM-ILD with pneumomediastinum (Zhou et al., 2020). Moreover, liver dysfunction is an extra-muscular sign in patients with anti-MDA5 positive CADM (Nagashima et al., 2019), which might explain why these elevated cytokines were associated with ALT and AST. Thereby, these phenomena support our results to some extent.

We further studied the receptors of the differentially expressed cytokines by published GEO datasets and found IL18R1 was increased in the skin, blood and muscle tissues of IIMs. IL18, as a pro-inflammatory cytokine, stimulates Th1 cells to produce IFN-γ (Nakanishi et al., 2001). IL18 receptor, IL18R, is primarily composed of two subunits, IL18R1 and IL18RAP. Under the action of IL18, these two subunits show a high affinity and form a complex, thus activating intracellular signaling pathways, such as NF-κB and AP-1 transcription factors (Yasuda et al., 2019). Previous studies have shown that an increased serum IL18 correlated with ILD in DM (Gono et al., 2010), and that IL18 was expressed in muscle infiltrating cells of patients with myositis (Helmers et al., 2018). Moreover, endothelial cells, smooth muscle cells and CD8^+^ cells expressed a high content of IL18R (Tucci et al., 2006). The possible reason for this is that the activation of interferon by the IL18 related signaling pathway plays an important role in IIMs.

In addition, flow cytometry results first showed that the number of IL18R1^+^ CD4^+^ cells in anti-MDA5 positive DM patients was increased, while that of IL18R1^+^ CD4^+^ cells was decreased. To the best of our knowledge, there are no reports on the relationship between IL18R1 and anti-MDA5 positive DM. Generally, Th1 cells producing IFN-γ stimulate classically activated macrophages (Mosmann and Coffman, 1989). In DM-ILD, activated Th1-type pulmonary T cells are closely associated with steroid-resistant DM-IP (Kurasawa et al., 2002). Given that anti-MDA5 positive DM patients tend to develop ILD with a poor prognosis, this may be responsible for the increased proportion of Th1-related cytokines and IL18R1^+^ CD4^+^ cells in the peripheral blood of patients. Another study revealed that exhausted CD8^+^ T cells can downregulate the IL18 receptor, thereby reducing the reactivity to bacteria co-infections and cytokines (Ingram et al., 2011). This might explain why anti-MDA5 positive DM patients are more susceptible to lung infection.

There are some limitations of this study. Firstly, this study was a retrospective study. Therefore, all clinical data were obtained from medical records, some of which might be incomplete. Secondly, the classification of IIMs was performed using Bohan and Peter’s criteria (Bohan and Peter, 1975), instead of the 2017 classification criteria (Lundberg et al., 2017). Thirdly, we found that the expression level of IL18R1 was of significance in IIMs, but the potential mechanism remains to be explored in the future.

In this study, we explored the serum cytokine profiles in different IIMs MSA groups, including anti-ARS positive, anti-MDA5 positive, and anti-TIF1γ positive IIMs, and their clinical correlations. Furthermore, we demonstrated that IL18R1 was of significance in both muscle tissue and peripheral blood in IIMs, which revealed that the role of IL18 and its receptor might engage in the pathogenesis of IIMs.

## Data Availability

The datasets presented in this study can be found in online repositories. The names of the repository/repositories and accession number(s) can be found in the article/Supplementary Material.

## Glossary

IIMs: Idiopathic inflammatory myopathies
DM: dermatomyositis
PM: polymyositis
IBM: inclusion body myositis
ADM: amyopathic dermatomyositis
MSAs: myositis-specific antibodies
MDA5: melanoma differentiation gene 5
TIF1γ: transcription intermediary factor 1-γ
ARS: aminoacyl transfer RNA synthetases
SRP: signal recognition particle
ILD: interstitial lung disease
RP-ILD: rapidly progressive interstitial lung disease
CK: creatine kinase
ALT: Glutamic-pyruvic transaminase
AST: glutamic-oxalacetic transaminase
HDL: high density lipoprotein
LDL: low density lipoprotein
LDH: lactate dehydrogenase
ESR: erythrocyte sedimentation rate
CRP: C-reactive protein
HRCT: high-resolution CT
MMT8: manual muscle testing 8
CTACK: cutaneous T cell-attracting chemokine
Eotaxin: a CC chemokine selective for Eosinophils
IL1Rα: IL-1 receptor antagonist
IL7: interleukin 7
IL18: interleukin 18
IP10: interferon gamma induced protein 10
MCP1: monocyte chemoattractant protein-1
MCP3: monocyte chemoattractant protein-3
MCSF: macrophage colony-stimulating factor
MIG: monokine induced by interferon (IFN)-gamma
SCGFβ: stem cell growth factor β
