# Supplementary material for: The Expression of Cytokine Profiles and Related Receptors in Idiopathic Inflammatory Myopathies
Source: Front Pharmacol. 2022 Apr 20;13:852055. doi: 10.3389/fphar.2022.852055 (PMC9065407; doi:10.3389/fphar.2022.852055)
Supplement: Supplementary file 1 [file Table1.docx]

**Supplement table:**

Table: The follow-up patients and treatments in 93 IIMs patients

|  | n=93 |
| --- | --- |
| Follow-up |  |
| 3 months | 18 |
| 6 months | 14 |
| 12 months | 7 |
| Treatments during follow-up |  |
| Glucocorticoids | 92 (98.9) |
| Tacrolimus | 50 (53.8) |
| Cyclophosphamide | 21 (22.6) |
| Hydroxychloroquine | 13 (14.0) |
| Thalidomide | 8 (0.1) |
| Intravenous immunoglobulin | 7 (0.1) |
| Methotrexate | 4 (0.0) |
| Plasmapheresis | 2 (0.0) |
| Mycophenolate mofetil | 2 (0.0) |
| Azathioprine | 2 (0.0) |
| Tofacitinib | 1 (0.0) |
